# Clinical Use of Aided Cortical Auditory Evoked Potentials as a Measure of Physiological Detection or Physiological Discrimination

**DOI:** 10.1155/2012/365752

**Published:** 2012-10-08

**Authors:** Curtis J. Billings, Melissa A. Papesh, Tina M. Penman, Lucas S. Baltzell, Frederick J. Gallun

**Affiliations:** ^1^National Center for Rehabilitative Auditory Research, Portland Veterans Affairs Medical Center, Portland, OR 97239, USA; ^2^Department of Otolaryngology/Head & Neck Surgery, Oregon Health & Science University, Portland, OR 97239, USA; ^3^Department of Speech and Hearing Sciences, Indiana University, Bloomington, IN 47405, USA

## Abstract

The clinical usefulness of aided cortical auditory evoked potentials (CAEPs) remains unclear despite several decades of research. One major contributor to this ambiguity is the wide range of variability across published studies and across individuals within a given study; some results demonstrate expected amplification effects, while others demonstrate limited or no amplification effects. Recent evidence indicates that some of the variability in amplification effects may be explained by distinguishing between experiments that focused on physiological detection of a stimulus versus those that differentiate responses to two audible signals, or physiological discrimination. Herein, we ask if either of these approaches is clinically feasible given the inherent challenges with aided CAEPs. N1 and P2 waves were elicited from 12 noise-masked normal-hearing individuals using hearing-aid-processed 1000-Hz pure tones. Stimulus levels were varied to study the effect of hearing-aid-signal/hearing-aid-noise audibility relative to the noise-masked thresholds. Results demonstrate that clinical use of aided CAEPs may be justified when determining whether audible stimuli are physiologically detectable relative to inaudible signals. However, differentiating aided CAEPs elicited from two suprathreshold stimuli (i.e., physiological discrimination) is problematic and should not be used for clinical decision making until a better understanding of the interaction between hearing-aid-processed stimuli and CAEPs can be established.

## 1. Introduction

The potential clinical benefits of a measure of brain encoding and plasticity in hearing aid users have driven a growing interest in aided cortical auditory evoked potentials (CAEPs). A better understanding of the effects of hearing aids on brain function, and resulting behavior, may improve the current science underlying successful rehabilitation of hearing loss. CAEPs, a type of event-related electroencephalography (i.e., scalp-recorded electrical brain activity) recorded 50–300 ms following stimulus onset, are thought to reflect neural activity in reverberant thalamocortical circuits (for a review see [1, 2]). Aided CAEPs, or potentials recorded when stimuli are presented via a hearing aid, have been proposed as a possible physiological measure of the effects of amplification on the brain. Many studies have explored the potential use of aided CAEPs, demonstrating considerable variability in results across experiments and individual participants [3–21]. The variability across studies highlights the current uncertainty surrounding the clinical usefulness of aided CAEPs. 

Methodological differences contribute to the variable results that exist in the aided CAEP literature. Much of the existing aided CAEP literature can be grouped into two general approaches: (1) a focus on physiological response detection, or (2) emphasis on physiological response discrimination. The physiological detection approach compares the CAEPs from an (inaudible or barely audible) unaided stimulus, to the response obtained from the same stimulus that has been processed by a hearing aid and delivered at a suprathreshold level. In this case, the unaided CAEP is often absent or weak, while the aided CAEP is often present with robust waveform morphology. The absence or presence of a response demonstrates a good correlation with inaudible and audible stimuli, amounting to a physiological correlate of detecting the presence of sound. Historically, CAEPs have successfully been used to estimate behavioral thresholds (approx. within 10 dB of behavioral threshold) of both normal-hearing and hearing-impaired populations [22–24]. However, it remains to be established whether such strong correlations between physiological and behavioral thresholds are maintained in the case of aided CAEPs, in which the stimuli have been altered by hearing aid processing.

In contrast to the physiological detection approach, the physiological discrimination approach compares CAEPs from two audible stimuli to determine differences between the waveforms (e.g., unaided versus aided conditions in normal-hearing individuals or two audible aided conditions with varied parameters). Physiological discrimination is measured by specific differences in waveform morphology (i.e., differences in peak latencies and peak amplitudes) between two present waveforms. 

**Figure 1:**
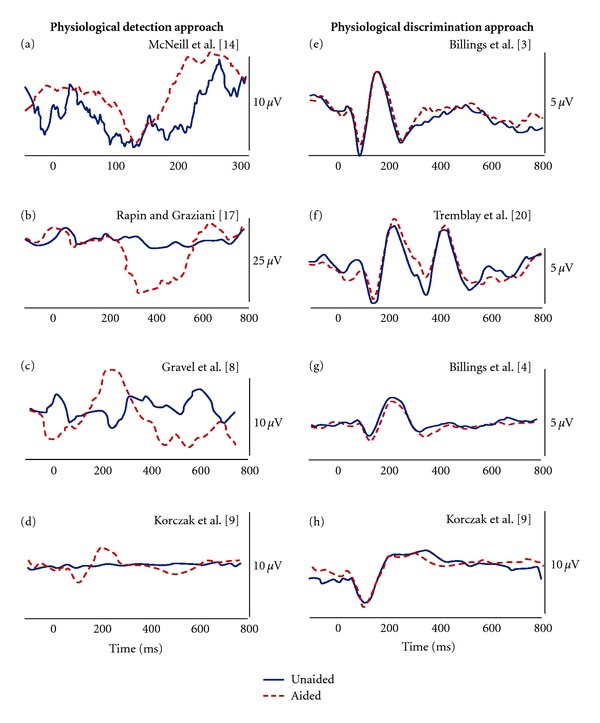
Examples of physiological detection (a–d) and physiological discrimination (e–h) approaches from the aided CAEP literature. Results across these studies demonstrate significant amplification effects (unaided versus aided) for physiological detection, but very limited amplification effects for physiological discrimination. All figures were modified from published figures; the appropriate citation is indicated for each panel (see Table 1 for details).

The different focus of these two approaches contributes to the variability in the existing literature involving aided CAEPs. Significant changes in waveform morphology (i.e., amplification effects) were often found when individuals/groups were tested in studies that used a physiological detection approach, comparing inaudible to audible conditions (e.g., [8, 14, 17]); whereas, amplification effects were often absent or small in studies that used a physiological discrimination approach comparing two suprathreshold responses (e.g., [3, 4, 20]). A comparison of these approaches is presented in Figure 1 and the corresponding Table 1, where the examples in the left column (a–d) demonstrate clear amplification effects obtained in a physiological detection approach; in contrast, examples of a physiological discrimination approach displayed in the right column (e–h) show small or absent amplification effects. It is noteworthy that many of the early publications highlighted case studies, and that despite the significant number of aided CAEP publications, only a limited subset displayed electrophysiological waveforms.

In addition to considering the audibility of the signal, it is equally important to take into account the audibility of the underlying noise and its relationship to the signal (i.e., signal-to-noise ratio or SNR). SNR is a key contributor to both unaided and aided CAEP morphology [4, 25]. Accounting for SNR is particularly important when establishing the effects of hearing aids on auditory processing because hearing aids both contribute circuit noise inherent to signal processing and because hearing aids amplify ambient environmental noise. When establishing the clinical utility of aided CAEPs, it is important to establish the effects of noise on CAEPs in situations where signal and noise are audible in both unaided and aided conditions or when two different aided conditions are being compared. Clinicians might encounter problems when fitting a hearing aid if they assume that changes to the hearing aid should improve the morphology of the evoked response when in reality no change should occur.

The purposes of this study are to characterize the existing aided CAEP literature relative to two potential clinical approaches, and to determine whether these approaches (i.e., physiological detection and physiological discrimination) demonstrate clinical utility for encoding hearing-aid-processed signals. Specifically, we asked two questions:

1. Will aided CAEPs be different for a near-threshold signal relative to a suprathreshold signal (i.e., physiological detection)?
2. Will aided CAEPs be different for two suprathreshold signals (i.e., physiological discrimination)?

We set out to answer these questions in the aided CAEP domain by recording hearing aid output and eliciting CAEPs with the recorded stimuli. 

## 2. Methods

CAEPs were recorded using hearing-aid-processed stimuli. In addition, a background noise masker was presented to the normal-hearing participants as a means of simulating the audibility factors that are present when an individual with hearing impairment is fit with a hearing aid. Testing noise-masked normal-hearing participants allowed tight control of audibility (i.e., audibility of hearing-aid-processed signals and hearing aid noise) while avoiding hearing-impairment-related confounds associated with recording CAEPs from individuals with hearing impairment. 

### 2.1. Participants

Twelve individuals (six women and six men) participated in the study (mean age = 22.1 years; SD = 2.47). All participants were right handed with normal hearing from 250 to 8000 Hz (≤20 dB HL), were in good general health, reported no significant history of otologic or neurologic disorders, and denied use of mood or sleep-altering medications. All participants provided informed consent, and all research complied with the regulations of the Portland Veterans Affairs Medical Center Institutional Review Board.

### 2.2. Stimuli

#### 2.2.1. Hearing Aid Signal and Noise

Hearing-aid-processed stimuli were used to elicit CAEPs. Signals were 1000-Hz tones recorded from the output of two hearing aids: Hearing Aid A was a currently available digital hearing aid, Hearing Aid B was the same analogue hearing aid used in previous studies from our laboratory [3, 4]. The frequency response of both hearing aids was matched in the test box of a hearing aid analyzer (Fonix 8000, Frye Electronics, Tigard, Oregon) to be within 3 dB at frequencies between 200 and 5000 Hz using a 45 dB pure-tone sweep. The hearing aids were then placed on the right ear of the Brüel and Kjær Head and Torso Simulator (type 4128C) using stock foam earmolds with no venting. The Head and Torso Simulator was placed inside an Eckel Corp. fully anechoic chamber (reverberation time constant of 2 ms and background noise level of –10 dB SPL at 1000 Hz). A 450-ms, 1000-Hz tone with 9 ms rise/fall times and an interstimulus interval (ISI) of 1900 ms was presented via a Cambridge Soundworks free-field speaker placed at 0 degrees azimuth at a distance of 1.5 meters from the hearing aid microphones. Volume control wheels, available noise cancellation algorithms, and directional microphones were deactivated on both hearing aids. Overall hearing aid gain and speaker output level were then varied systematically to result in three recordings (see Table 2). Electroacoustic analysis of the settings used in the three recordings revealed that attack and release times for Hearing Aid A were 5 ms and 225 ms, respectively; using a 1000-Hz tone, compression ratios were measured at 1.25 : 1 from 50–75 dB and 1 : 1 from 75–90 dB. Hearing Aid B attack and release times were 2.5 ms for both recordings; a 1 : 1 compression ratio was found from 50–60 dB and 2 : 1 from 60–90 dB for Recording 2 settings, and an essentially linear input/output function was measured for Recording 3 settings.

 The goal was to record three signals that varied in signal-to-noise ratio (SNR). As indicated in Table 2, resulting SNRs for the three recordings were 8 dB, 11 dB, and 23 dB. Recordings were approximately two minutes in length and consisted of continuous hearing aid noise and a total of 50 signal presentations. The use of an anechoic chamber for the recordings ensured that the hearing aid noise consisted entirely of circuit noise, as the environmental noise was negligible (−10 dB SPL in the 1/3 octave band surrounding 1000 Hz). The three recordings were then scaled in Matlab (Version 7.0, Mathworks, Natick, MA) to create stimuli of varying absolute levels such that the audibility of the tonal signal and the underlying hearing aid noise varied systematically relative to the background noise masker (see Section 2.2.2 for details of background noise creation). 

**Figure 2:**
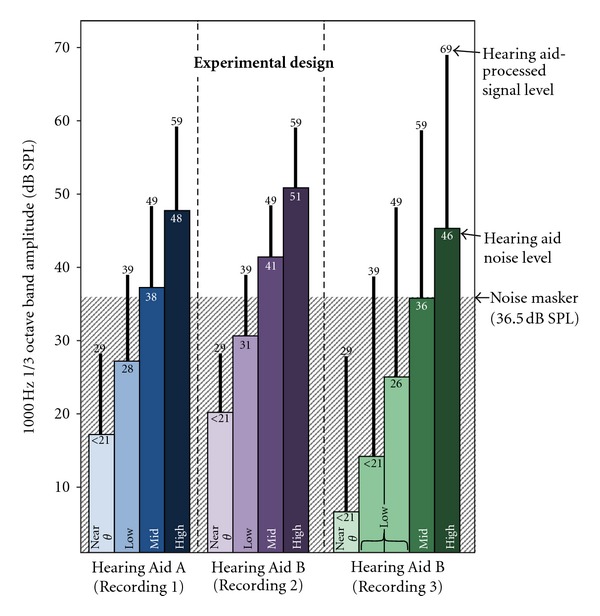
Experimental design. One-third octave band levels at 1000 Hz are shown for the three hearing-aid-processed recordings. Scaling of the recordings resulted in Near *θ*, Low, Mid, and High conditions. The shaded background shows the background noise masker level relative to hearing aid signal and noise levels.

A schematic depicting the hearing aid signal (i.e., 1000-Hz tone) and noise levels in relation to the background noise masker for each hearing aid condition is shown in Figure 2. For low-input recordings from Hearing Aid A and Hearing Aid B, four scaling factors were calculated that adjusted the level of the tone and hearing aid noise in approximately 10 dB steps to produce the following presentation levels: Near *θ*, a near-threshold level at which both tone and hearing aid noise were inaudible due to the noise masker amplitude (intended to represent an inaudible unaided condition); Low, a level at which the signal was above the level of the noise masker but the hearing aid noise was below; Mid and High, two levels at which the signal and hearing aid noise were both audible but absolute signal level differed. Due to the higher ratio of signal level to hearing aid noise obtained in the higher input level recording from Hearing Aid B, five stimulus levels were necessary in order to span the range of audible and inaudible signals. In total, 13 experimental conditions were presented to each participant. Notice that, within each block of hearing aid conditions, the SNR between the signal level and the hearing aid noise remains constant (i.e., approx. 11 dB for Hearing Aid A (Recording 1), 8 dB for Hearing Aid B (Recording 2), 23 dB for Hearing Aid B (Recording 3), while the overall stimulus output level changes in 10 dB steps. However, the effective SNR was much smaller for Near *θ* and Low conditions due to the background noise masker. Levels displayed in Figure 2 are 1/3 octave band values centered at 1000 Hz measured with a Brüel and Kjær 2260 Investigator sound level meter fitted with a Brüel & Kjær ear simulator (number 4157).

**Figure 3:**
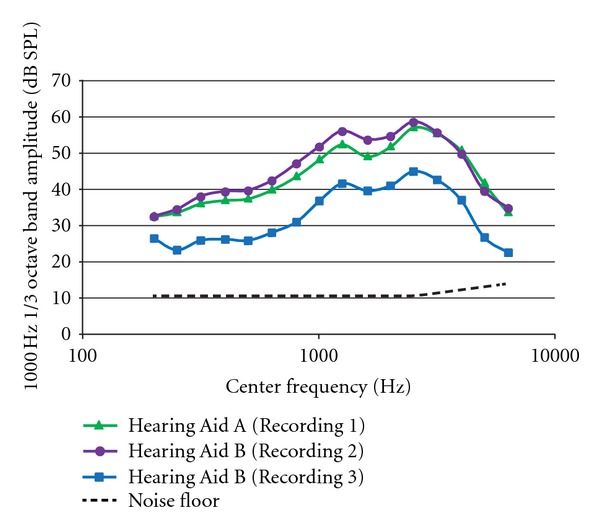
Frequency spectra of hearing aid noise for each of the three hearing aid conditions. Values are 1/3 octave bands with center frequencies between 200 and 6300 Hz. Hearing aid noise was measured for the 59-dB signal level condition for each recording. The general pattern of noise spectra is similar across conditions with a spectral peak at 1000 Hz, the frequency of the signal. The noise floor of the measurement system is shown with the dashed line (note: the lower limit of the sound level meter was 10.5 dB).

 The hearing aid noise spectra obtained from the three 59 dB hearing aid condition presentations are represented in Figure 3 (measured with the sound level meter in 1/3 octave bands with center frequencies from 100 to 6300 Hz) along with the noise floor of the measurement system. To ensure that the lowest signal level presentations were below threshold, participants were asked to listen to each of the three lowest level conditions and report whether or not they could detect the tonal signal. This measure confirmed that the lowest stimulus levels for each of the three hearing aid recordings were either inaudible or barely audible to all participants.

#### 2.2.2. Background Noise Masker

In order to simulate hearing-impaired thresholds and to control the audibility of the hearing-aid-processed signal and underlying hearing aid noise, a continuous background noise masker was created in Matlab passing a Gaussian white noise through a series of 1/3 octave band filters with center frequencies from 100 to 5000 Hz. The output of the filters was adjusted to generate a noise masker with a spectrum matching the thresholds of a patient with a moderate, sloping hearing loss. The spectrum of the noise masker was verified with a spectrum analyzer in 1/3 octave bands. In addition, behavioral thresholds at octave and interoctave frequencies from 250 to 8000 Hz were established using ER-3A (Etymotic Research, Inc., Elk Grove Village, IL) insert earphones. The thresholds of four participants were established using 1 dB steps in a 1-up, 2-down procedure while the background masker was played through the audiometer. Mean behavioral thresholds for the four individuals were 11.0, 15.75, 22.25, 24.25, 28.0, 34.75, 44.75, 43.25, and 8.75 dB HL at frequencies of 250, 500, 750, 1000, 2000, 3000, 4000, 6000, and 8000 Hz, respectively.

### 2.3. Electrophysiology

For each of the 13 conditions, a recorded 50-tone wav file was repeated three times, yielding a total of 150 tone presentations recording over approximately six minutes for each condition. Both the noise masker and hearing aid noise were continuous throughout each block of trials, and all stimuli were presented in the right ear using an ER-3A insert earphone and the Stim2 system (Compumedics Neuroscan, Charlotte, NC). The presentation order of the three hearing aid conditions was randomized across subjects, and the various stimulus levels were randomized within each hearing aid condition to minimize order effects across participants. Two-minute listening breaks were given between each condition, and subjects were offered a longer break after one hour of testing. Acquisition sessions lasted three hours and consisted of consenting, audiometric testing, electrode placement, and CAEP acquisition. Participants were seated comfortably in a double-walled sound attenuating booth, and they were instructed to ignore the auditory stimuli and to watch a closed-captioned movie of their choice. 

 Evoked potential activity was recorded using an Electro-Cap International, Inc. cap which housed 64 tin electrodes. The ground electrode was located on the forehead and Cz was the reference electrode. Data were rereferenced offline to an average reference. Horizontal and vertical eye movement was monitored with electrodes located inferiorly and at the outer canthi of both eyes. The recording window consisted of a 100-ms prestimulus period and a 700-ms poststimulus time. Using Scan 4.5 (Compumedics Neuroscan, Charlotte, NC), evoked responses were analog bandpass filtered online from 0.15 to 100 Hz (12 dB/octave roll off) and converted using an analog-to-digital sampling rate of 1000 Hz. Trials with eye-blink artifacts were corrected offline using Neuroscan software. This blink reduction procedure calculates the amount of covariation between each evoked potential channel and a vertical eye channel using spatial, singular value decomposition and removes the vertical blink activity from each electrode on a point-by-point basis to the degree that the evoked potential and blink activity covaried [26]. After blink correction, trials containing artifacts exceeding 70 *μ*V were rejected from averaging. After artifact rejection, the remaining sweeps were averaged and filtered offline from 1 Hz (highpass filter, 24 dB/octave) to 30 Hz (lowpass filter, 24 dB/octave). Averages for 98% of conditions tested had more than 100 accepted trials; the remaining 2% had between 70 and 100 accepted trials. N1 and P2 peak amplitudes and latencies were determined by agreement of two judges. Each judge used temporal electrode inversion, global field power (GFP) traces, and even and odd sweep waveform versions (to demonstrate replication) for a given condition. Peaks in the Near *θ* conditions were very difficult to identify because of the electrophysiological noise. Therefore, in order to quantify synchrony in the near-threshold conditions, the rectified area was measured in the time region from 40 to 300 ms as a representation of synchrony of the P1-N1-P2 complex. Area values were generated at all Near *θ* and Low conditions. 

### 2.4. Statistical Analysis

We fit the linear mixed model representation of the repeated measures analysis of variance (ANOVA). This model has the two-fold advantage of (1) being fit using maximum likelihood so that all observations are included in the analysis and not just observations for subjects with complete data, and (2) taking into account nonsymmetrical variances that may occur across conditions. Main effects of hearing aid condition were tested for two contrasts: Low versus Mid and Mid versus High. The Mid versus High comparison directly tests the physiological discrimination approach while the Low versus Mid comparison verifies that when SNR is changing, the aided CAEP is also likely to change. Where main effects were found, post-hoc comparisons were made for each hearing aid condition. 

To test the effectiveness of the physiological detection approach, paired comparisons were completed on rectified area measures of Near *θ* versus Low for the three hearing aid conditions. Area measures were used because of the large number of absent responses in the Near *θ* condition. 

## 3. Results

The current study investigated the ability of aided CAEPs to demonstrate amplification effects using both neural physiological response detection and physiological discrimination approaches. Based on a review of literature, we hypothesized that aided CAEPs would show the most robust effect of amplification in neural response detection approaches which correlate with the difference in response to audible versus inaudible stimuli. In contrast, aided CAEP discrimination approaches that reflect the ability of the auditory system to represent differences between audible hearing-aid-processed stimuli were expected to show weak amplification effects. Results, presented below, are organized to address these two primary hypotheses.

### 3.1. Physiological Detection

**Figure 4:**
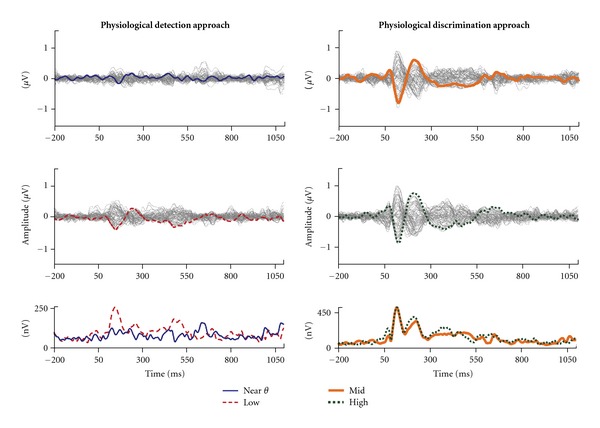
Grand average (*n* = 12) butterfly plots and global field power (GFP) waveforms of physiological detection and physiological discrimination results. Waveforms are collapsed across hearing aid recordings. Near *θ* (top left), Low (middle left), Mid (top right), and High (middle right) conditions are displayed with the Cz-electrode highlighted. Bottom panels show overlaid comparisons for Near *θ* versus Low conditions and Mid versus High conditions. Robust differences are shown for physiological detection (bottom left) and minimal differences are shown for physiological discrimination (bottom right).

**Figure 5:**
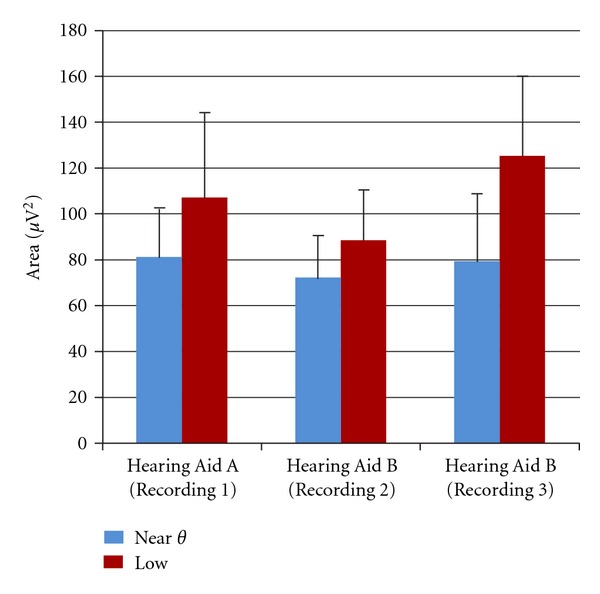
Area measurements for Near *θ* and Low conditions for three recordings. Low conditions yielded higher area values than Near *θ* conditions for all three hearing aid recordings, demonstrating aided CAEP morphology differences that are present for physiological detection.

This study addressed the effects of amplification on audible and inaudible stimuli by comparing the Near *θ* condition (i.e., aided CAEP responses to the lowest stimulus level at which the tone level was at or below the noise masker level) to the Low condition (i.e., aided CAEP responses to the stimulus level at which the tone was just audible above the noise masker). For Hearing Aid B (Recording 3), two different Low conditions were used; however, to simplify the data analysis, all data for the two Low conditions were averaged together to result in a measurement for one Low condition. Butterfly plots (overlaid responses at all electrodes across the scalp) and the global field power plots (a quantification of simultaneous activity across the scalp; [27]) calculated from the grand average for these two stimulus levels across subjects and hearing aid conditions are presented in Figure 4 (left). The butterfly plot of the Near *θ* condition is shown in the top-left panel with the response of the Cz electrode highlighted in blue. The butterfly plot of the Low condition response is shown in the center-left panel with the Cz electrode highlighted in dashed red. The bottom-left panel shows the GFP waveforms for the Near *θ* and Low conditions overlaid in blue and dashed red, respectively. Notice that the response to the Near *θ* condition contains considerable noise across electrodes. In contrast, responses to the Low condition result in visible peaks in activity across electrodes resulting in clearly identifiable peak waves in the GFP plot as well as the Cz electrode response. N1 and P2 peaks were difficult to identify in many of the Near *θ* conditions, resulting in large amounts of missing data (approx. 50% of N1 and P2 peaks were not able to be identified), making statistical analysis using traditional peak latency and amplitude values difficult; therefore, an area measure (rectified area was calculated from 40 to 300 ms) was used to provide an overall measure of synchrony in the P1-N1-P2 region of the waveform.  Figure 5 displays area values for the Near *θ* and Low conditions for all three hearing aid conditions. Paired comparisons between Near *θ* and Low conditions generally demonstrated significantly higher areas for Low conditions relative to Near *θ* conditions: 

Hearing Aid A (Recording 1): *t* = −2.077, *df* = 11, *p* = .062; 

Hearing Aid B (Recording 2): *t* = −2.853, *df* = 11, *p* = .016; 

Hearing Aid B (Recording 3): *t* = −4.225, *df* = 11, *p* = .003.

### 3.2. Physiological Response Discrimination

The discrimination task of the current study measured the ability of aided CAEP measures to reflect differences between two clearly audible stimuli (i.e., Mid and High conditions). To demonstrate the main effect of signal level, butterfly and GFP plots were constructed from grand average responses across subjects and hearing aid recordings for Mid and High conditions (Figure 4, right). The top-right panel of Figure 4 depicts the butterfly plot in response to the Mid conditions with the Cz electrode highlighted in orange. Notice that a clear response waveform is present and the coherence between responses among electrodes on the butterfly plot. The center-right panel shows the butterfly plot in response to High conditions with the Cz electrode highlighted in dotted green. Again, a response is clearly present as expected given audibility of the signal. In the lower-right panel, GFP responses for the Mid and High conditions are overlaid and plotted in orange and dotted green, respectively. Notice that the GFP response between the two conditions is very similar with nearly identical peak amplitudes and latencies. This figure helps to highlight the difficulty in using aided CAEP measures to establish the brain's ability to physiologically discriminate between two suprathreshold hearing-aid-processed stimuli.

**Figure 6:**
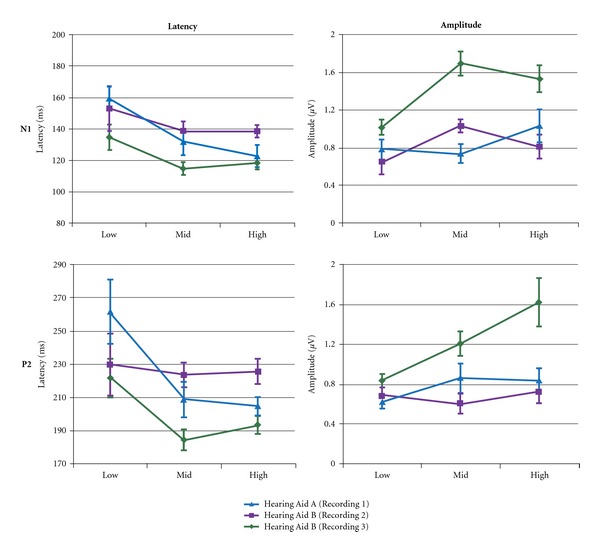
Mean latency and amplitude measures for Low, Mid, and High conditions as a function of hearing aid recording (error bars: standard error of the mean). Generally, a change from Low to Mid conditions results in decreases in latency and increases in amplitude, and a change from Mid to High results in minimal change in latency and amplitude.

 N1 and P2 peak amplitude and latency data for the Low, Mid, and High conditions are displayed in Figure 6 with corresponding statistical analyses shown in Table 3. As mentioned above, the responses to the two Low conditions for Hearing Aid B (Recording 3) were averaged together for latency/amplitude comparisons between the three hearing aid conditions. The results indicate no main effect of Mid versus High conditions for N1 and P2 measures. However, comparisons of Low versus Mid conditions resulted in main effects of N1 and P2 latency and amplitude. 

### 3.3. SNR Effects on CAEPs

Results from previous literature indicate that CAEP responses are more sensitive to changes in SNR than to changes in absolute signal level [4, 25]. In the present study, a lack of significant change in N1 and P2 amplitudes or latencies in response to the Mid and High conditions supports the idea that waveforms generally do not change when SNR is held constant. To further quantify the effects of SNR on aided CAEPs, we compared responses across Low, Mid, and High conditions. The Low conditions had smaller SNRs than the Mid and High conditions because of the audible background noise masker. For both the Mid and High conditions in all three hearing aid conditions, SNR was dictated by the constant ratio of signal level to hearing aid noise within the 1000-Hz band surrounding the signal tone. The SNR for each hearing aid condition was 11 dB for Hearing Aid A (Recording 1), 8 dB for Hearing Aid B (Recording 2), and 23 dB for Hearing Aid B (Recording 3). Therefore, the SNR was constant at the two highest presentation levels within each hearing aid condition but varied between hearing aid conditions. If SNR drives amplitude and latency changes in CAEPs, we would expect to see significant changes between the Low and Mid/High conditions, and potentially differences between hearing aids due to differences in SNR. The amplitude and latency plots (Figure 6) generally indicate the expected changes in N1 amplitudes and latencies. 

This visual impression is confirmed by the significant main effect of Low to Mid level conditions on N1 and P2 amplitudes and latencies, which is not found in the comparison between Mid to High level conditions (Table 3). Further, the effect of the difference in SNR between hearing aid conditions was apparent when comparing the statistical significance between the Low and Mid level stimuli for each hearing aid condition. The hearing aid condition with the largest output SNR (Hearing Aid B, Recording 3) was most likely to show significant differences in N1 and P2 amplitudes and latencies between Low and Mid level recordings, followed by the hearing aid with the second largest output SNR (Hearing Aid A, Recording 1). The hearing aid condition with the poorest SNR (Hearing Aid B, Recording 2) was least likely to demonstrate significant differences in peak amplitudes or latencies. Overall, these findings corroborate earlier reports of the significant influence of SNR on CAEP recordings. 

## 4. Discussion and Conclusions

The purpose of this study was to help clarify some of the variability that is seen in decades of aided CAEP research. This variability is shown in Figure 1/Table 1 where some studies demonstrate robust effects of amplification (a–d) while others do not (e–h). We hypothesized that large portions of this variability can be explained by whether the signal and underlying noise are audible relative to the contrasting condition. Two approaches, physiological detection (i.e., the absence versus presence of a CAEP) and physiological discrimination (i.e., the differentiation of two present responses), were tested. Figure 4 demonstrates the overall results of this study relative to these two approaches. Physiological detection demonstrates robust amplification effects, while physiological discrimination demonstrates limited differences between Mid and High conditions. These results are in agreement with those found by Korczak and colleagues [9] where both approaches can be identified in subsets of their data. Korczak et al. compared unaided and aided CAEPs in two groups of individuals with hearing loss (some with moderate hearing loss and some with severe hearing loss) using stimuli presented at approximately 70 and 85 dB HL. They found improved CAEPs only for the lower level stimulus or when the unaided stimulus was near threshold (i.e., physiological detection; Figure 1(d)). Interestingly, when both unaided and aided stimuli were likely above threshold (i.e., physiological discrimination), as was the case for the moderate hearing-impaired group using a 85-dB stimulus, limited effects of amplification were found on N1 and P2 waves (Figure 1(h)). 

### 4.1. Clinical Feasibility: Physiological Detection versus Physiological Discrimination

**Figure 7:**
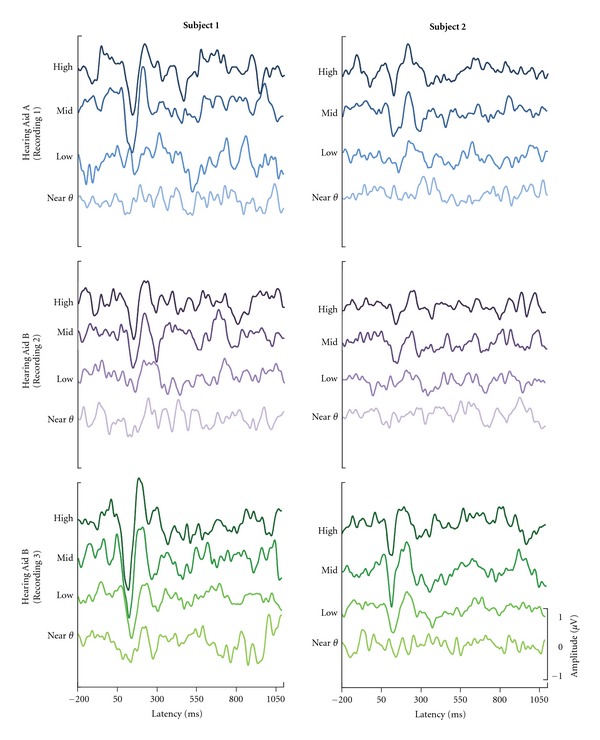
Cz-electrode waveforms for two representative individuals across the three hearing aid recordings. For both participants, the Near *θ* condition shows an absent or very small response, while the Low condition shows a more pronounced response. Mid and High responses are present and similar to each other. Effects of hearing aid recording are somewhat apparent with the most robust waveforms occurring in the Hearing Aid B (Recording 3) condition.

The physiological detection approach appears to be a reasonable use of aided CAEPs because these measures are sensitive to differences in detectability of an inaudible or barely audible signal and a suprathreshold signal. Our results, and the results of other past studies, demonstrate robust amplification effects when taking a detection approach [8, 9, 14, 16, 17]. This study simulates the process a clinician may use in fitting a hearing aid, in which hearing aid gain is increased in 10-dB steps and the resulting CAEP is examined. In this scenario, the increasing signal level demonstrates a robust effect.  Figure 7 shows two representative individuals from the 12 participants and demonstrates the clinical process of increasing the gain of a hearing aid. The Near *θ* curve shows an absent response in most cases; whereas, the Mid and sometimes Low conditions show present responses. 

 In contrast, these data and examples from the literature [3, 4, 9, 15, 20] demonstrate that approaching aided CAEPs from a physiological discrimination perspective is problematic, especially if background noise is audible, as is the case for the Mid and High conditions in this study. For two individuals (Figure 7), similarities between Mid and High conditions are apparent and demonstrate the difficulty in differentiating between the CAEPs to two suprathreshold signals. Importantly, statistical differences were found for comparisons between Low and Mid conditions (see Table 3); in these cases, differences reflect changes in SNR as the hearing aid noise is not yet audible. Therefore, the comparison between Low and Mid conditions can be considered a successful example of physiological discrimination. Indeed, CAEPs have been used successfully as a measure of physiological discrimination for decades; however, our understanding of *aided* CAEPs is still lacking. When evoking CAEPs with hearing-aid-processed stimuli, it remains unclear when a discrimination approach is valid; it may be valid only for specific hearing aids, specific hearing aid settings, specific stimuli, or as in this study, specific conditions (e.g., Low versus Mid and not Mid versus High). Certainly, clinical decisions based on a physiological discrimination approach would be premature. Additional research is needed to delineate what hearing aid and stimulus interactions are affecting the evoked response. 

**Figure 8:**
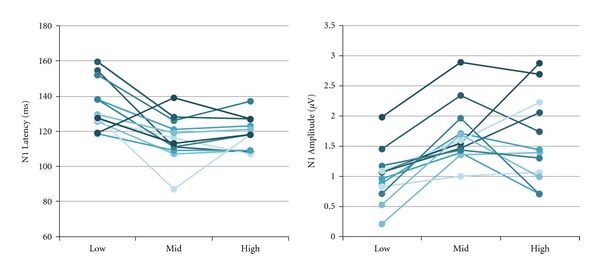
Individual N1 latency and amplitude values demonstrating the physiological discrimination approach. Low, Mid, and High conditions are shown for Hearing Aid B (Recording 3). The general trends, consistent with Figure 6, show considerable changes from Low to Mid conditions and minimal changes from Mid to High conditions. Variability across individuals is evident with some individuals contradicting the general trends.

 It is important to consider subject factors as well. The audibility of a broadband stimulus and the underlying noise will vary depending on the hearing configuration of the individual being tested. The participants in this study were young normal-hearing individuals, and a noise masker was used to simulate thresholds that were comparable with a typical sloping hearing loss. However, even with tightly controlled audibility and a pure tone stimulus, variability across participants was found.  Figure 8 demonstrates how 10-dB increments in signal level affect N1 latency and amplitude in the 12 individuals tested. Testing hearing-impaired individuals with broadband stimuli would likely result in increased variability across participants because of varying etiologies of hearing loss and differences in threshold across frequencies. Therefore, use of aided CAEPs in individuals in a clinical setting using a physiological discrimination approach is likely to result in considerable variability resulting from the many varying subject and stimulus factors.

It should be noted that the method of scaling used in the design of this study to modify signal level is different than clinical hearing aid gain adjustments in that modification of gain can lead to a wide range of acoustic modifications to the signal. While SNR has been shown to remain similar across gain settings in some hearing aids [4], SNR can also vary significantly from one device and recording condition to another [28]. These considerations are important when using a physiological discrimination approach; while one hearing aid may show a physiological amplification affect, another device may not because of the specific acoustic features that are modified. This variability in outcomes severely limits the clinical usefulness of physiological discrimination approaches in aided CAEPs until more is understood about the interaction between hearing-aid-processed signals and their effects on the evoked response.

### 4.2. Major Acoustic Contributors to Aided CAEPs

As mentioned above, the problems related to the physiological discrimination approach likely result from a combination of subject and stimulus factors. SNR and onset modification are two stimulus characteristics whose importance has been demonstrated in the literature (e.g., [3, 4, 12, 25, 29–31]). First, the effects of SNR are important to consider when recording evoked potentials at the level of the cortex, because cortical neurons are more sensitive to SNR than to absolute signal level [32, 33]. The audibility of underlying noise in the hearing-aid-processed signal, whether amplified ambient noise or circuit noise, must be considered when interpreting aided CAEPs. Second, onset changes to the time waveform that result from hearing aid processing are also important. The N1-P2 CAEP is an onset response, meaning that it is generated when many cortical pyramidal cells fire synchronously to the onset of a stimulus. These neurons are especially sensitive to abrupt changes in amplitude or frequency; therefore, hearing-aid modifications to the onset are important to consider [12, 30]. These considerations are complicated when speech stimuli are used, because it becomes difficult to determine the SNR across different portions of the speech signal, particularly in light of changes in compression across running speech. 

The results of this study seem to indicate that the contribution of stimulus factors can be minimized when the physiological detection approach is used. Specific signal-processing modifications made by the hearing aid are less important when the comparison response waveform is absent. In contrast, subtle acoustic changes (e.g., modification of SNR or onset characteristics) are essential when comparing two audible signals in a physiological discrimination approach. To characterize acoustic changes, it is necessary to complete in-the-canal recordings of hearing-aid-processed signals. Only then can measures of the important signal modifications be made and related to the resulting aided CAEPs. 

### 4.3. Conclusions

Two approaches for using aided CAEPs, physiological detection and physiological discrimination, were tested to determine the clinical usefulness of each. Results are in agreement with an analysis of the literature (see Figure 1), and they demonstrate that physiological detection, or a determination of the presence of a response to an audible signal relative to the absent response of an inaudible signal, is likely a valid use of aided CAEPs and provides an indication of the encoding of the aided signal at the level of the auditory cortex. In contrast, the physiological discrimination approach (i.e., the comparison of waveforms that are generated by two audible signals) can be problematic and difficult to interpret in individuals when using hearing-aid-processed stimuli. A more detailed understanding of how hearing aid processing modifies stimulus acoustics (e.g., SNR and onset characteristics) is needed before the physiological discrimination approach should be used for clinical decision making.

## Figures and Tables

**Table 1:** Outline of the experimental conditions used in the studies cited in Figure 1. Note that the parameters listed below are specific to the conditions used to generate the waveform data presented in Figure 1 and do not necessarily represent all conditions presented in each study. The eight studies included represent aided CAEP data in the literature for which unaided and aided waveforms were able to be reproduced.

	Subjects			Experimental design				Source
	*n*	Characteristics	Stimulus	Duration (ms)	ISI (ms)	Signal level	Conditions
Figure 1 Detection approach examples								
(a) McNeil et al., 2009 [14]	1	68 yo male; severe to profound SNHL	/ba/	115	750*	60 dB nHL	Unaided and aided	Figure 1, page 81: unaided and left hearing aid
(b) Rapin and Graziani, 1967 [17]	1	21 mo female; rubella, sedated	500-Hz tone	Not specified	Not specified	109 dB re: 0.0002 dynes/cm^2^	Unaided and aided	Figure 4, page 892: 109 dB with and 109 dB without aid
(c) Gravel et al., 1989 [8]	1	7 mo male; severe to profound SNHL	/da/	Not specified	Not specified	Not specified	Unaided and aided (aid set to user settings)	Figure 3, page 271
(d) Korczak et al., 2005 [9]	7	Adults; severe to profound SNHL	/ba/ and /da/	150	950	80 dB ppeSPL	Unaided and aided (aid set to MCL)	Figure 3, page 176: Standard responses in lower panel, left
Figure 1 Discrimination approach examples								
(e) Billings et al., 2007 [3]	13	Young adults; normal hearing	1000-Hz tone	757	1910	50 dB SPL	Unaided and aided (20 dB gain)	Figure 2, page 238: 50 dB aided and 50 dB unaided waveforms
(f) Tremblay et al., 2006 [20]	7	Young adults; normal hearing	/si/	655	1910	64 dB SPL	Unaided and aided (average gain of 19 dB)	Figure 7, page 99: Top panel
(g) Billings et al., 2011 [4]	9	Young adults; normal hearing	1000-Hz tone	756	1910	40 dB SPL	Unaided and aided (gain of 20 dB)	Figure 6, page 7: Panel (a) unaided waveform, panel (b) aided waveform
(h) Korczak et al., 2005 [9]	4	Adults; moderate SNHL	/ba/ and /da/	150	950	85 dB HL	Unaided and aided (aid set to MCL)	Figure 3, page 176: Standard responses in lower panel, center

*Reference does not state whether this value refers to onset to onset, or offset to onset. All other interstimulus intervals (ISIs) refer to offset to onset.

**Table 2:** Three recordings of hearing aid output. Specific characteristics of the three hearing aid recordings used in this study to elicit aided CAEPs.

	Hearing Aid	Gain at 1000 Hz^1^	Input^2^	Output SNR^3^
Recording 1	A	30 dB SPL	25 dB SPL	11 dB
Recording 2	B	30 dB SPL	25 dB SPL	8 dB
Recording 3	B	10 dB SPL	45 dB SPL	23 dB

^1^Gain with a 45 dB SPL input (electroacoustically verified).

^2^Input: input to hearing aid microphones in the sound field.

^3^Output SNR: difference between hearing-aid-processed signal and noise at 1000 Hz (1/3 octave band).

**Table 3:** Statistical anlalysis. A linear mixed model representation of the repeated measures ANOVA resulted in a main effect of the level contrast with post-hoc comparisons where the main effect was significant.

Conditions	Main effect			Hearing aid A (Recording 1)			Hearing aid B (Recording 2)			Hearing aid B (Recording 3)		
	*F *Value	*df*	*p*	*F *Value	*df*	*p*	*F *Value	*df*	*p*	*F *Value	*df*	*p*
Low to Mid level												
N1 Latency (ms)	9.36	3,97	<0.0001	13.78	1,97	0.0003	4.03	1,97	0.0474	10.25	1,97	0.0018
P2 Latency (ms)	17.53	3,96	<0.0001	30.52	1,96	<0.0001	0.47	1,96	0.4955	21.6	1,96	<0.0001
N1 Amplitude (*μ*V)	6.92	3,97	0.0003	0.03	1,97	0.868	4.18	1,97	0.0435	16.54	1,97	<0.0001
P2 Amplitude (*μ*V)	3.16	3,96	0.0282	2.84	1,96	0.0954	0.67	1,96	0.4134	5.96	1,96	0.0164
Mid to High level												
N1 Latency (ms)	0.64	3,97	0.5894	—	—	—	—	—	—	—	—	—
P2 Latency (ms)	0.38	3,97	0.7698	—	—	—	—	—	—	—	—	—
N1 Amplitude (*μ*V)	0.31	3,97	0.8163	—	—	—	—	—	—	—	—	—
P2 Amplitude (*μ*V)	2.07	3,96	0.109	—	—	—	—	—	—	—	—	—

## Abbreviations

CAS: Central auditory system
SNR: Signal-to-noise ratio
SD: Standard deviation
GFP: Global field power
CAEPs: Cortical auditory evoked potentials
ANOVA: Analysis of variance.
